# On the Merits and Demerits of the Ovarian Section

**Published:** 1852-09

**Authors:** 


*On the Merits and Demerits of the Ovarian Section.*—The following are the conclusions which the "British and Foreign Medico-Chirurgical" Reviewer arrives at after a careful consideration of the whole of the cases bearing upon this point, although they must only be considered on the whole as approximations to truth:

1st. That in any case in which it is considered advisable to remove an ovarian tumor, it is justifiable to make a small preliminary incision into the abdomen, for the purpose of determining whether the tumor be inherent or not.

2d. If the tumor be adherent, the incision is to be immediately closed entirely, or to such an extent as merely to leave an aperture the size of that made by an ordinary trocar, and we may then expect that this operation will not, on the average, be followed by much more fatal results than common tapping.

3d. That where the tumor consists of a simple cyst or cysts, with but small solid deposit, it may be extirpated with as good a chance of success as attends the performance of the more serious surgical operations, and with the further prospect of the cure remaining permanent.

4th. The existence of much solid deposit, or of extensive adhesions, absolutely forbids the operation, which should be brought to a termination immediately on the discovery of either.

Finally, we may add our belief, that the plan proposed by Mr. Wilson, of tying each bleeding vessel separately, so as to dispense with the ligature round the pedicle is an important improvement; and that, if experience shows that it is sufficient for the cure of the disease to remove only a part of the cyst, the operation will be rendered considerably more hopeful.—*Brit. and For. Med. Rev.*

